# Attention training for infants at familial risk of ADHD (INTERSTAARS): study protocol for a randomised controlled trial

**DOI:** 10.1186/s13063-016-1727-0

**Published:** 2016-12-28

**Authors:** Amy Goodwin, Simona Salomone, Patrick Bolton, Tony Charman, Emily J. H. Jones, Andrew Pickles, Emily Robinson, Tim Smith, Edmund J. S. Sonuga-Barke, Sam Wass, Mark H. Johnson

**Affiliations:** 1 Department of Child and Adolescent Psychiatry, Institute of Psychiatry, Psychology and Neuroscience (IoPPN), King’s College London, London, UK; 2 Centre for Brain and Cognitive Development, Birkbeck, University of London, London, UK; 3 MRC Social, Genetic and Developmental Psychiatry Centre, IoPPN, King’s College London, London, UK; 4 NIHR Biomedical Research Centre at South London and Maudsley NHS Trust, London, UK; 5 Department of Psychology, IoPPN, King’s College London, London, UK; 6 Department of Biostatistics, IoPPN, King’s College London, London, UK; 7 Department of Psychological Sciences, Birkbeck, University of London, London, UK; 8 Department of Psychology, University of Southampton, Southampton, UK; 9 Department of Experimental Clinical and Health Psychology, Ghent University, Ghent, Belgium; 10 School of Psychology, University of East London, London, UK; 11 Department of Psychology, University of Cambridge, Cambridge, UK

**Keywords:** ADHD, Attention, Infancy, Cognitive training, Early intervention, Familial risk

## Abstract

**Background:**

Attention deficit hyperactivity disorder (ADHD) is a prevalent neurodevelopmental disorder that can negatively impact on an individual’s quality of life. It is pathophysiologically complex and heterogeneous with different neuropsychological processes being impaired in different individuals. Executive function deficits, including those affecting attention, working memory and inhibitory control, are common. Cognitive training has been promoted as a treatment option, based on the notion that by strengthening the neurocognitive networks underlying these executive processes, ADHD symptoms will also be reduced. However, if implemented in childhood or later, when the full disorder has become well-established, cognitive training has only limited value. INTERSTAARS is a trial designed to test a novel approach to intervention, in which cognitive training is implemented early in development, before the emergence of the disorder. The aim of INTERSTAARS is to train early executive skills, thereby increasing resilience and reducing later ADHD symptoms and associated impairment.

**Methods/design:**

Fifty 10–14-month-old infants at familial risk of ADHD will participate in INTERSTAARS. Infants will be randomised to an intervention or a control group. The intervention aims to train early attention skills by using novel eye-tracking technology and gaze-contingent training paradigms. Infants view animated games on a screen and different events take place contingent on where on the screen the infant is looking. Infants allocated to the intervention will receive nine weekly home-based attention training sessions. Control group infants will also receive nine weekly home visits, but instead of viewing the training games during these visits they will view non-gaze-contingent age-appropriate videos. At baseline and post treatment, infant attention control will be assessed using a range of eye-tracking, observational, parent-report and neurophysiological measures. The primary outcome will be a composite of eye-tracking tasks used to assess infant attention skills. Follow-up data will be collected on emerging ADHD symptoms when the infants are 2 and 3 years old.

**Discussion:**

This is the first randomised controlled trial to assess the potential efficacy of cognitive training as a prevention measure for infants at familial risk of ADHD. If successful, INTERSTAARS could offer a promising new approach for developing early interventions for ADHD.

**Trial registration:**

International Standard Randomised Controlled Trial registry: ISRCTN37683928. Registered on 22 June 2015.

**Electronic supplementary material:**

The online version of this article (doi:10.1186/s13063-016-1727-0) contains supplementary material, which is available to authorized users.

## Background

Attention deficit hyperactivity disorder (ADHD) is a neurodevelopmental disorder with an estimated prevalence of 3–5% of the general population [1, 2]. Characterised by inattention, hyperactivity and impulsivity [3], ADHD significantly impacts on everyday functioning in multiple domains, with knock-on effects on quality of life [4]. Although ADHD is typically diagnosed, and its treatment initiated, in middle childhood, prospective longitudinal studies suggest that there are prodromal behavioural, cognitive and attentional markers prior to the emergence of the full disorder, which in some cases are evident at the age of 3 years [5]. Even in the early stages the condition is associated with significant impairment [6], but the problems often escalate as children grow, giving rise to substantial difficulties in later life [7–9]. In a recent prospective study it was estimated that high levels of preschool ADHD symptoms were associated with a 17-fold increase in economic burden to the mental health, education and criminal justice systems by early adulthood [10].

Typically initiated in middle childhood, a multimodal combination of medication and psychological therapies are recommended as treatment for ADHD on the basis of the best available trial evidence [11]. However, evidence of developmental continuity from early childhood, and the potential for ADHD-related impairments to escalate over time, has prompted the development of early intervention as an alternative treatment strategy [12]. Medication is not regarded as a viable early treatment option in all but the severest cases – being less efficacious and associated with more adverse events when implemented during the preschool period [13]. There is strong resistance from many parents and clinicians to the use of medication with preschool children. The most frequently used nonpharamacological intervention for preschool children with ADHD is parent training. In parent training, parents are taught to modify their child’s behaviour using techniques derived from social learning theory principles [14]. However, trial data using blinded outcomes suggests that parent training has only limited effects on core ADHD symptoms, but it does have other positive effects on children’s behaviour more generally [15, 16].

Although not yet implemented as part of an early intervention or prevention strategy, cognitive training has recently been promoted as a treatment more generally for ADHD [17]. Cognitive training aims to exploit the brain’s plasticity to improve the function of the brain networks thought to mediate the risk for ADHD. It does this through controlled exposures to cognitively demanding tasks over multiple training sessions [18]. To date cognitive training approaches for ADHD have targeted executive functions, including those affecting attention, working memory and inhibitory control, known to be impaired in a substantial proportion of children with ADHD [18, 19]. Taken as a whole, results from randomised controlled trials of cognitive training for ADHD have been disappointing so far. Two recent meta-analyses of randomised controlled trials [15, 18] found that, as with parent training, reductions in ADHD symptoms were not found on well-blinded outcomes. In fact, for working memory training, the most studied approach, overall meta-analytic effects were close to zero [18] despite promising initial findings [20].

While these results are disappointing, the logic underpinning cognitive training as a treatment for ADHD is consistent with it being considered as a candidate for early, or preventative, intervention. Indeed, it is possible that the reason it has not worked so far is that it is being implemented too late in development, when deficits are already established and more resistant to change, while at the same time brain plasticity is potentially decreasing. Furthermore, there is evidence that atypicalities in attention may develop very early on in individuals who later develop symptoms of ADHD, i.e. during the first year of life. In longitudinal studies, young children with greater symptoms of inattention and hyperactivity at 3 years of age have been shown to exhibit shorter eye-fixations on a screen-based task [21], and shorter epochs of focussed attention in a naturalistic context [22] during infancy. This indicates continuities in the developmental pathway, from executive attention skills in infancy, to emerging symptoms of ADHD in the preschool years. Accordingly, it may be crucial for cognitive training to be implemented as early as is feasible during development – perhaps even prior to the emergence of early signs of the disorder.

INTERSTAARS is a departure from previous cognitive training approaches for ADHD as it represents the first attempt to use cognitive training to target and strengthen executive control networks in infancy with individuals *at risk,* but not yet manifesting, the later development of ADHD. The logic is that in infancy, when neural plasticity is potentially greater and the brain is more amenable to positive environmental effects, far-transfer effects may be more likely [23]. Consistent with this logic, a recent systematic review concluded that cognitive training targeted at younger individuals led to significantly more widespread transfer of training effects [23].

Previous research with typically developing infants suggests that this age group is responsive to appropriate cognitive training. Wass et al. [24] used a gaze-contingent training paradigm to target attention control in typical 11-month-old infants. Using eye-tracking technology, infants viewed animated games on a screen, and the stimuli they were presented with in each of the games changed adaptively, contingent on where on the screen they were looking. The games were designed to target a number of aspects of executive attention including sustained attention and inhibitory control. Following four training sessions, infants showed training effects on a battery of cognitive tasks when compared to an untrained, active control group [24].

In order to test the potential efficacy of cognitive training as a preventative approach for ADHD, it is necessary to identify a group of infants who are at an increased risk of developing the disorder. Fifty infants who have a first-degree relative (either a parent or an older sibling) with ADHD will take part in the INTERSTAARS trial. The risk of ADHD is known to be familial, with elevated prevalence in individuals who have a parent or sibling with the disorder. Sibling recurrence rates have been documented to be between 12 and 35% [25–27], and similar recurrence rates have been found between parents and children [26].

Cognitive training for infants has previously been tested in a laboratory setting [24]. However, administering the training in a research laboratory can be a burden for families because they have to travel to the laboratory for each training session. This burden may be particularly large for at-risk families, such as families where a parent or an older sibling also has ADHD. Therefore, to optimise the acceptability of the intervention for families, the training programme will be carried out in families’ homes. The feasibility of implementing the training games outside of a research laboratory has previously been established in both community centre [28] and school [29] settings.

The main objectives of the INTERSTAARS trial are to test:

1. Whether a home-based attention control training programme will improve attention control in infants at familial risk of ADHD
2. Whether these effects are mediated by changes in the executive attention system and will transfer to changed attentional behaviours in a range of testing contexts
3. Whether these changes in attention control are associated with the amelioration of emerging ADHD symptoms at 2 and 3 years of age

## Methods/design

### Study design and setting

INTERSTAARS is a two-site, double-blind, phase 2 randomised controlled trial, involving clinicians and researchers in London (Centre for Brain and Cognitive Development, Birkbeck University of London and the Institute of Psychiatry, Psychology and Neuroscience, King’s College London) and Southampton (Department of Psychology, University of Southampton), United Kingdom. The trial is embedded within the Studying Autism and ADHD Risks (STAARS) project at the Centre for Brain and Cognitive Development (CBCD), Birkbeck. STAARS is a longitudinal observational study following infants from 5 months to 3 years of age, with the aim of identifying early predictors of ADHD and autism spectrum disorder [30].

### Eligibility and recruitment

Fifty 10–14-month old infants with a first-degree relative (parent or older sibling) with a clinical or probable diagnosis of ADHD will take part in the INTERSTAARS trial. Eligible families must: (1) live within a 2-h travel distance from either London or Southampton, (2) have at least one parent/caregiver who is fluent in English and, (3) have agreed to take part in the affiliated STAARS project. Participants will hear about the trial via a variety of routes including: the National Health Service (South London and Maudsley, and Solent Trusts), recruitment databases at the University of Southampton, Institute of Disorders of Impulse and Attention (the South Hampshire ADHD Register and the Programme for Early Detection and Intervention), adult ADHD clinics, health visitors, community paediatricians, ADHD support groups and charities. Posters advertising the study will also be placed in local libraries, cafes, children’s centres, general practitioner (GP) surgeries, play centres, nurseries and relevant online forums. Where there is a suspected case of ADHD within a family (parent or sibling) a formal assessment using age-appropriate standardised screening questionnaires will be used to establish the probable existence of ADHD^1^.

### Exclusion criteria

Infants who meet any of the following criteria based on parent-report will be excluded from the trial: (1) serious medical or developmental conditions such as epilepsy, heart conditions, cerebral palsy, intellectual disability, (2) significant uncorrected vision or hearing problems, (3) significant prematurity (less than 36 weeks’ gestation), (4) genetic conditions such as Down’s syndrome or Fragile X syndrome and (5) the eye-tracking technology used throughout the trial cannot successfully track the infant’s eyes after four attempts at the baseline assessment.

### Participant timeline

**Fig. 1:**
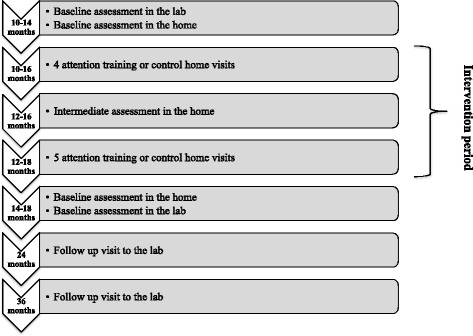
Summary of participant timeline

Parents who express an interest in taking part in the trial will be contacted by a research assistant at the relevant trial site who will give them further information about the study in person, over the phone or by email. All interested families will be given at least a 24-h window in which to consider their participation in the study. A summary of the timeline for participants is shown in Fig. 1. For families who agree to take part in the trial, baseline and outcome assessments will be conducted both in the laboratory at the CBCD, Birkbeck, and in the participant’s home. Baseline assessments will be conducted when the infant is enrolled between 10 and 14 months of age (in the home and in the laboratory). Outcome assessments will be conducted following the intervention period, when the infant is 14–18 months of age (in the home and in the laboratory). In addition, an intermediate outcome assessment will be conducted mid-way through the intervention period (home only). Baseline and outcome assessments will include a series of behavioural, neurophysiological, eye-tracking and parent-report measures, all of which have been previously tested with infants (see ‘Outcomes’ below).

**Fig. 2:**
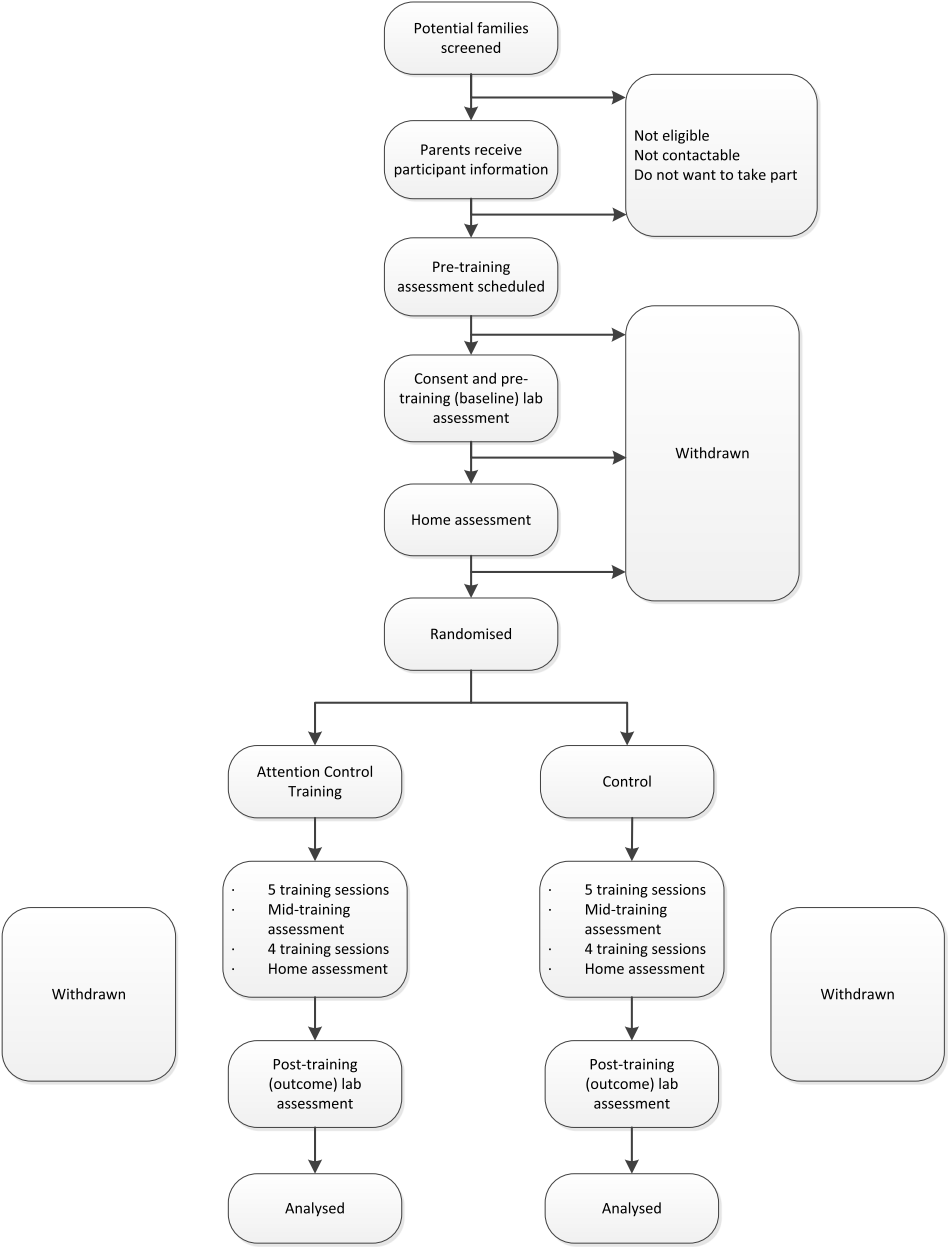
Consolidated Standards of Reporting Trials (CONSORT) flow diagram

Parents’ written consent will be received at their first visit to the CBCD before any baseline measures have been collected. All costs incurred by participants’ visits to the laboratory, e.g. travel and meals will be reimbursed. Participants will attend follow-up visits at the laboratory when they are 24 and 36 months of age. For further details on the participant timeline, please refer to the trial’s Consolidated Standards of Reporting Trials (CONSORT) flow diagram [31] in Fig. 2.

### Intervention

Infants allocated to the intervention group will receive nine home-based attention training sessions, to be carried out on a weekly basis. The attention training programme uses gaze-contingent animations that operate via eye-tracking technology to target attention control. Infants view animated games on a computer screen and where they look on the screen determines what they see. Each training session involves two trained researchers visiting the family’s home and setting up the home-based eye-tracking equipment used to administer the training. Data will be collected using a 60-Hz Tobii X2-60 eye-tracker. Each training visit will last for approximately 1 h. During training sessions, the infant sits on their parent’s lap, and the games are presented to them on a Dell 19-inch monitor screen, with a screen resolution of 1024 × 768. The games run using MATLAB scripts, written by author SW and previously tested with infants [24]. Games are intended to be attractive and enjoyable for infants. The games are adaptive, so that as the infant performs better, the levels increase and the games become more challenging. The original training programme [24] featured four games. In order to maintain engagement over the longer training phase planned for INTERSTAARS (nine versus four training sessions), additional versions of the original games have been added to the training programme, closely based on those used by Wass et al. [24] but with different surface features. Together with one new game, they are designed to train attention control across mixed cognitive domains that include sustained attention, working memory, visual search and inhibitory control. Five of the games are described below. The other four games included in the programme use the same training paradigm as one of those described, but with new graphics and sound effects to help maintain infant engagement.

#### Butterfly

In the Butterfly game, the infant is presented with a cartoon butterfly on the computer screen. When the infant fixates on the butterfly it flies across the screen, from left to right. As it flies, various distractors (clouds, trees, houses) scroll from left to right. If the child looks to any of the moving (and, therefore, highly likely to be visually salient, [32]) distractors, they disappear and only the butterfly remains on-screen. The child has to learn that the only way to progress on the task is to look to the butterfly and inhibit the prepotent urge to look to distractors. The salience of the distractors increases contingent with performance, i.e. the better performing infants are trained with more frequent and more salient distractors. Two of the other games included in the training programme use the same training paradigm as Butterfly, with new graphics and sound files.

#### Windows

In the Windows game, the infant is presented with two windows on the left and right of the computer screen. A cartoon earwig is presented in one window, together with an attention-getter (a red circle) and an audio sound effect to draw the infant’s attention to the earwig. Once the child has looked to the earwig, it disappears, and the infant is presented with a fixation point (a cartoon flower) in the centre of the screen. When the infant fixates on the flower it rotates until a delay period has elapsed, and then disappears. If the infant then looks to the correct window (where the earwig was previously displayed) they receive a reward sequence showing the earwig flying off the screen, accompanied by sound effects. As the infant progresses through the difficulty levels, the delay period becomes longer and the number of windows increases. Another game included in the training programme uses the same training paradigm as Windows, but with new graphics and sound files.

#### Stars

In the Stars game, the infant must search for the correct target within a complex visual scene. The target (a cartoon character in a brightly coloured star) is presented alongside eight distractors (e.g. planets, clouds). The infant must look to the target within a time limit. If they correctly locate the target they receive an animated reward sequence. As the infant progresses successfully through trials, the levels become more difficult by increasing the salience of the distractors.

#### Suspects

In the Suspects game, a target (a colourful cartoon elephant) is presented alongside a distractor. If the infant fixates on the target elephant within a certain time period then they receive a reward. A larger number of distractors are presented contingent on performance. Every 12 trials, the target switches: where previously the child has received a reward for looking to the elephant, they now start to receive a reward for looking to a chicken. The target changes periodically between the elephant and the chicken, changing every 12 trials.

#### Puzzle Memory

In the Puzzle Memory game, a cartoon character is presented in one of two locations. Following an animation, the character disappears and a fixation point is presented elsewhere on the screen. If the child subsequently looks back to the location where the character disappeared and maintains their gaze there, the character reappears. A second character is then presented in the second location, followed by the same sequence (character disappears, fixation point is presented). However, throughout this sequence, the previously found character remains on screen. During the response window, the infant has to use the stored memory to maintain their gaze on the location where the new character has disappeared, and inhibit the urge to look to the previously found character, which is more immediately salient. At higher difficulty levels, the number of locations increases. Another game included in the training programme uses the same training paradigm as Puzzle Memory but with new graphics and sound files.

To ensure consistency of delivery between researchers fixed rules have been developed for administering the training games. Six training games are played per session. Each training game is played for a maximum of 300 s, or until the infant becomes fidgety and has not been engaged with the game for 20 s or more. The MATLAB scripts are programmed to automatically recommend which game the researcher should play next during a training session, according to an order that is pseudorandomised across training visits. Cross-site quality control meetings will be held to assess treatment delivery at both sites using objective measures including: the duration of each training session, the number of games played, the duration of each game played, and the percentage of eye-tracking data collected during each session.

### The control arm

Infants in the control group will be allocated to a passive viewing condition. The procedure for infants in the control group will be identical to those in the intervention group, except that instead of viewing the attention training games during home visits they will view infant-friendly, non-gaze-contingent television clips for an equivalent amount of time. The set-up procedures, duration and audiovisual style of the clips will match the intervention condition as closely as possible. For justification of this control condition, please refer to the later ‘Discussion’ section.

The control condition runs using MATLAB scripts developed at the CBCD. Each control session involves two trained researchers visiting the family’s home and setting up the home-based eye-tracking equipment. Data will be collected using a 60-Hz Tobii X2-60 eye-tracker. During the session, the infant sits on their parent’s lap and the television clips are presented to them on a Dell 19-inch 5:4 aspect-ratio monitor screen with a resolution of 1024 × 768. Each television clip is scaled to full screen, with the aspect ratio maintained. Clips are played with their original audio. Frame rates for the clips range from 23 to 30 frames per second (FPS). Individual clips from the same television programme are combined into a ‘theme’. There are nine themes in total, similar to there being nine training games in the intervention. The same fixed rules used to deliver the intervention are also used to administer the control condition. Six themes are played per session. The MATLAB scripts automatically recommend which theme the researcher should play next, according to an order that is pseudorandomised across sessions. Each theme is played for a maximum of 300 s, or until the infant becomes fidgety and has not been engaged for 20 s or more. The same objective measures used to assess the delivery of the intervention, e.g. session duration, number of themes played, will also be collected for the control arm.

### Assignment of the intervention

#### Randomisation

Randomisation will be completed by a web-based service, at the King’s Clinical Trial Unit (KCTU, [33]). Randomisation will be done on an individual level, minimising by trial site (London or Southampton) and gender of the infant (male or female). Allocation is performed when a trained, unblinded researcher logs into the KCTU system, and the system then sends an automatic email to the researcher confirming the allocation of the participant. This procedure will be performed after the baseline assessments have been completed, before the first attention training/control session.

#### Blinding

Researchers who administer the baseline and outcome assessments will be blind to treatment condition. Efforts will be made to ensure that parents are also blind to treatment condition. To maintain parent blindness throughout the trial the number of home visits, contact with trainers, and set-up procedures will be identical between the intervention and control groups. At the end of the trial, a qualitative interview will be conducted with the primary parent/caregiver to assess whether they have remained blind to treatment condition throughout the intervention period. Due to the nature of the intervention, it will not be possible for the researchers who administer the attention training to remain blind to the treatment condition. However, the treatment and control conditions are run using automated MATLAB scripts and data metrics from these tasks will be analysed by different, blinded researchers. The researchers who administer the intervention will not be involved in the collection of outcome data. The only exception to this will be the intermediate outcome assessment. Researchers who conduct the intermediate outcome assessment will not be blind to treatment condition. To note, data from the intermediate assessment is not included as any of the primary or secondary outcomes.

#### Adherence and fidelity

Adherence to the trial will be judged to exist as a minimum of six training/control sessions per infant, with a minimum average session duration of seven min. Fidelity will be measured as the percentage of eye-tracking data collected across training/control sessions (i.e. the amount of time the infant was looking towards the screen and the eye-tracker was detecting their gaze).

### Outcomes

A summary of all measures collected in the trial can be found in Additional file 1: Table S1. Data for the primary and secondary outcomes will be collected before and after the intervention period, both in the laboratory and in the home. The infant’s parent will be present for all measures collected during these assessments. Parents are asked to respond to their infant’s needs but not to actively initiate interaction with their infant or try to direct their attention. Visits to the laboratory take approximately 5 h. Parents will be reminded at the start of each session that they can ask to stop or take a break at any time for any reason. All infant assessments will be videotaped whenever feasible.

#### Primary outcome

The primary outcome in the trial will be a composite of eye-tracking measures used to assess changes in infant attention control. This eye-tracking data will be collected in the home, using a 20-min eye-tracking battery designed to measure different aspects of infant attention. The tasks included in the battery are the: cognitive control task, sustained-attention task, sequence learning task, distractor task, visual paired comparison task, and gap-overlap task (see Table 1 for a description of each task). From this battery, three eye-tracking measures were selected for the primary outcome composite, based on their responsiveness to attention training in previous replications of the intervention [24]:

**Table 1:** Summary of eye-tracking battery

Task	Summary
*Cognitive control* adapted from [24, 49]	At the beginning of each trial, the infant is presented with a fixation point in the centre of the screen. Once the infant looks to the fixation point, an audio reward is presented, followed by a visual reward (a short animated clip), which is presented on either the left or right of the screen. The visual reward is presented on 1 side for 9 consecutive trials (pre-switch) before switching to the other side for the subsequent 9 trials (post-switch). Anticipatory saccades are coded based on the child’s looking behaviour during the anticipatory window (between the start of the auditory reward and the start of the visual reward). The dependent variable is the percentage of trials in which infants make a correct anticipatory saccade towards the location of the target stimuli in the pre- and post-switch phases.
*Sustained attention* adapted from [24]	The infant is presented with two ‘interesting’ (complex, detailed) and two ‘boring’ (noncomplex) static stimuli. For each stimulus, the experimenter records the length of the first 5 of the infant’s looks towards the stimulus presentation area. To qualify as a look the infant must visually engage with the stimulus for at least 1 s. To terminate the look, the infant must disengage from the stimulus for at least 1 s. The longest of the first 5 looks is termed the peak look duration. The dependent variable is the peak look duration towards the interesting stimuli.
*Gap-overlap* adapted from [47, 50]	The infant is presented with a stimulus in the centre of the screen. Once the infant fixates on this central stimulus, a peripheral stimulus appears on the left or right of the screen. When the infant moves their gaze from the central to the peripheral stimulus they receive an audiovisual reward. There are three conditions in this task: baseline, overlap and gap. In the baseline condition, the central stimulus disappears at the same time that the peripheral stimulus appears. In the gap condition, there is a 200-ms gap between the removal of the central stimulus and the appearance of the peripheral stimulus. In the overlap condition, the central stimulus remains on the screen after the peripheral stimulus appears. The dependent variable is the saccadic reaction time (ms) to move the eyes from the central to the peripheral stimulus in the overlap condition.
*Sequence learning* adapted from [51]	The infant is presented with a target cartoon character (e.g. a bunny rabbit) that can appear in one of 6 locations. If the infant moves their gaze to the correct target location prior to the emergence of the cartoon character then they receive an audiovisual reward. For the first 8 trials, the character appears in a sequence of locations 1, 3, 5. After 8 trials the sequence reverses. The dependent variable is the proportion of correct anticipatory saccades towards the target location.
*Visual paired comparison* adapted from [52]	The infant is presented with a static image (Image A) in the centre of the screen. Image A is presented until the infant has looked at it for a total of 10 s. Following this 10-s period, Image A is presented alongside a new image, Image B. The dependent variable is the infant’s look duration towards the new versus the familiar stimulus.
*Distractor* adapted from [53]	An animated cartoon is presented in the centre of the screen. Throughout its presentation, distractors randomly appear on the left or right side of the screen. If the infant looks towards the distractors then the cartoon is paused. The dependent variable is the number of looks towards the distractors.

1. Sustained attention (an infant’s ability to maintain their attention on a stimulus). This will be measured as the peak duration of infant looking time towards a series of interesting static stimuli in the sustained-attention task. Peak look duration is defined as the longest of the first five individual looks towards each stimulus
2. Disengagement effect (an infant’s ability to disengage their attention from a stimulus). This will be measured as the difference between mean saccadic reaction times between the baseline and overlap conditions in the gap-overlap task
3. Cognitive control (an infant’s ability to learn a rule, and later inhibit this information to shift attention to a new rule). This will be measured as the percentage of trials in which infants correctly anticipate the location of the target stimuli in the pre- and post-switch phases of the cognitive control task.

#### Secondary outcomes

Secondary outcomes were selected to assess whether changes in infant attention control following attention training transfer to more naturalistic contexts. The following measures will be secondary outcomes in the trial:

1. Parent-report measures of infant executive attention assessed by the Infant Behavior Questionnaire-Revised [34], including the effortful control, activity level and duration of orienting scales
2. Observational measures of infant attention to toys during toy play including the task orientation episode from the Laboratory Temperament Assessment Battery, Lab-TAB [35], and a structured free-play episode adapted from [36]. Behavioural coding will be completed using an in-house coding scheme and the Lab-TAB manual
3. Observational measures of infant social attention skills using the Early Social Communication Scales (ESCS) [37]. The following ESCS tasks will be administered: responding to the joint attention task, the social interaction task, the initiating joint attention task, and the book-reading task. Infant behaviours will be coded according to the ESCS manual
4. General attentiveness as measured by infant average look duration to the stimulus presentation area across the entire 20-min eye-tracking battery

#### ADHD symptom trajectories

Follow-up visits to the laboratory will be completed at 2 and 3 years of age. At age 3, standardised measures will be used to assess the presence of early ADHD symptoms including the Child Behaviour Checklist [38], the young child Diagnostic Interview Schedule for Children [39], and the teacher and parent-report Conners’ Early Childhood [40], as well as measures of executive function (the Behavioural Rating Inventory of Executive Functions [41]).

### Data collection and management

#### Sample size and power

Wass et al. [24] found an effect size of 0.69 on the cognitive control task following the attention training intervention in a sample of typically developing infants. Based on this effect, a sample of 50 participants (25 in each condition) is required to allow for sufficient power. This sample size will allow for 10% attrition (*n1* = 22, *n2* = 23), with 82% power and a two-tailed significance value of 0.05, using an analysis of covariance (ANCOVA) with baseline-outcome correlation of 0.6 (Stata 14 *sampsi,*) [42].

#### Data management

Parent-report measures will be stored using a secure online database called REDCap (Research Electronic Data Capture) [43]. To ensure blinding, only unblinded researchers will have access to the INTERSTAARS REDCap database. A subset of data (10%) will be double entered into REDCap to assess reliability of data entry across researchers. Experimental data (e.g. eye-tracking and neurophysiological measures) will be stored on a secure server at the CBCD.

#### Statistical methods

An intention-to-treat (ITT) approach will be used to analyse data from the trial, which will follow a Statistical Analysis Plan specified prior to data inspection, approved by the Data Monitoring and Ethics Committee, with analyses undertaken with uninformative treatment group labels. The primary outcome measure will be a composite of the three eye-tracking components selected above in ‘Outcomes’. The three components will be analysed together initially for evidence of a common treatment effect, equivalent to a treatment effect for the simple sum of normalized standardized scores of each component. A multivariate ANCOVA will then be used to test the treatment effect of the attention training group compared to the control group, using Stata *sem* [42].

#### Missing data

Missing baseline and outcome data will be treated as ignorable under the assumption of missing-at-random. This assumption can be made due to the repeated measures set-up in which both baseline and outcome variables are treated as dependent variables with all valid scores for each component included.

#### Sensitivity analysis

If more than 10% of participants are missing components of the primary outcome then a sensitivity analysis will be performed. This analysis will be completed using Stata *mi*.

### Ethical issues

#### Ethical approval

Ethical approval has been granted for the trial from the National Health Service Integrated Research Application Service, London Central Research Ethics Committee (Reference: 15/LO/0407).

#### Data monitoring

The Data Monitoring and Ethics Committee will meet biannually to discuss the INTERSTAARS trial. To monitor for possible adverse side effects of participating in INTERSTAARS, the following information will be collected regularly throughout the trial:

1. Infant sleep: we will use a Sleep Diary (developed by our own laboratory) to measure infant sleeping patterns. Data collected includes the amount of sleep during the day and during the night, and the number of times the infant wakes during the night. The Sleep Diary will be completed for the night before, and the night following, each attention training or control session
2. Infant fussiness: we will use a Fussiness Scale (developed by our own laboratory) to measure infant fussiness. Fussiness will be rated on a 1 to 5 scale for the 4 h following each attention training or control session, and for the 4 h that is comparable from the previous day, i.e. if a training session finishes at 2 p.m., fussiness will be rated from 2 p.m. to 6 p.m. for that day, and from 2 p.m. to 6 p.m. for the previous day.
3. The Standard Protocol Items: Recommendations for Interventional Trials (SPIRIT) checklist [44] for this manuscript is included as Additional file 2.

### Funding

The INTERSTAARS trial is funded by the charity MQ: Transforming Mental Health. Funding for follow-up visits for the study at 24 and 36 months of age is from the Medical Research Council (MRC), which funds the STAARS programme into which the INTERSTAARS trial is integrated.

## Discussion

INTERSTAARS is a two-site randomised controlled trial investigating the potential efficacy of nine weekly attention training sessions to improve attention control in infants at familial risk of ADHD. The trial is novel; cognitive training has not previously been applied to infants at increased risk of developing attention problems. While early psychological interventions have been championed as a way of reducing the risk for ADHD, studies have so far focussed on young children already displaying ADHD symptoms. Through its implementation in infancy, before symptoms have emerged, INTERSTAARS offers a potential preventative approach for infants at heightened risk. The intervention is home-based, making it a viable option for at-risk families. Results from the INTERSTAARS trial will make a novel contribution to research on early interventions for ADHD, and will help to further our understanding of infant attention in general.

While the trial will greatly contribute to these fields of research, potential limitations of the study should also be recognised. One difficulty encountered in the design of the trial was selecting an appropriate control condition. There were several options for control participants including: (1) treatment as usual, (2) a yoked control design and (3) a passive viewing condition. Treatment as usual was not selected because we wanted to ensure that differences between the control condition and the treatment condition were specific to attention training, and were not the result of another aspect of home visits, such as frequent interaction with researchers, or the amount of screen time. Furthermore, as INTERSTAARS targets an at-risk cohort (rather than a population already diagnosed, or exhibiting, ADHD symptoms) it is unlikely that the infants involved in the trial would have been previously receiving any form of intervention for ADHD prior to entering the trial.

In the second option for the control condition, a yoked control design, participants would view the attention training games, but the games would not be gaze-contingent. This condition appeared optimal in minimising the differences between the two conditions. However, pilot studies by SW showed that infants became bored quickly when watching the yoked control condition, making it difficult to match the conditions on overall screen time, so this condition was also not selected. Instead, a passive viewing condition was used. This is the same control condition used in previous studies with this attention training intervention [24, 28]. It allows for the conditions to be matched on screen time. However, the surface audiovisual features of what the infants view on the screen will differ between conditions.

There are also some limitations associated with using a familial risk group as the sample for the trial. Using a prospective design allows researchers to target a group of individuals who are at an increased risk of developing the disorder of interest, and to study this group in depth as they develop through infancy and beyond [45, 46]. Fifty infants at familial risk of ADHD will be recruited to the trial, a sample size that is modest enough to assess group differences following attention control training. However, it should be noted that not all of the infants included in the trial would develop ADHD without intervention. To generalise any findings to infer that attention control training leads to reduced ADHD recurrence will not be possible within this early stage trial. If significant group effects are found for attention control training in the INTERSTAARS sample then it will be important to test this approach with larger groups of infants in the future.

In addition to using larger samples, future research should also investigate the potential efficacy of attention training with other risk groups. ADHD is a heterogeneous disorder, in that different subgroups of the population may enter the disorder via different developmental pathways [47]. While the results of the INTERSTAARS trial will be relevant for infants at increased familial risk of ADHD, whether the results are also applicable to other groups of infants at risk of ADHD, e.g. premature infants [45], will require further investigation. If successful across ADHD risk groups, future research could investigate the impact of attention training on generic attention control skills, for individuals in the general population with subclinical markers of low attention control during infancy or for other developmental disorders that exhibit impaired attention control. This would be applicable to numerous other risk groups, including infants at risk of autism spectrum disorder [48]. The home-based nature of the intervention allows for it to be feasibly administered to high-risk populations who might otherwise be difficult to reach.

### Trial status

The first participant was enrolled and randomised in November 2015. Recruitment is ongoing.

## Additional files

Additional file 1: Table S1. Summary of measures. (PDF 99 kb)
Additional file 2: SPIRIT Checklist [44]. (PDF 106 kb)

## Abbreviations

ADHD: Attention deficit hyperactivity disorder
ANCOVA: Analysis of covariance
BASIS: British Autism Study of Infant Siblings
CBCD: Centre for Brain and Cognitive Development
ESCS: Early Social Communication Scales
FPS: Frames per second
INTERSTAARS: Intervention in STAARS
KCTU: King’s Clinical Trials Unit
Lab-TAB: Laboratory Temperament Assessment Battery
REDCap: Research Electronic Data Capture
STAARS: Studying Autism and ADHD Risks project

## Footnotes

1 Standardised questionnaires will be administered over the phone with the parent by a trained researcher. For siblings < 6 years of age, a shortened version of the Conners Early Childhood [40] will be administered. For siblings >= 6 years of age, a shortened version of the Conners 3 [54] will be administered. Cut offs for inclusion will be the presence of 4 (Conners Early Childhood) or 5 (Conners 3) DSM-5 ADHD symptoms on either the hyperactivity/impulsivity or inattention scale, and a positive score on the impairment scale. For parents, a shortened version of the Conners Adults ADHD Rating Scale (CAARS) [55] will be administered. Cut offs for inclusion using the CAARS will be the presence of 5 DSM-5 ADHD symptoms on either the hyperactivity/impulsivity or inattention scale.
