# Erratum to: Attention training for infants at familial risk of ADHD (INTERSTAARS): study protocol for a randomised controlled trial

**DOI:** 10.1186/s13063-017-2167-1

**Published:** 2017-09-11

**Authors:** Amy Goodwin, Simona Salomone, Patrick Bolton, Tony Charman, Emily J.H. Jones, Luke Mason, Andrew Pickles, Emily Robinson, Tim Smith, Edmund J.S. Sonuga-Barke, Sam Wass, Mark H. Johnson

**Affiliations:** 1 Department of Child and Adolescent Psychiatry, Institute of Psychiatry, Psychology and Neuroscience (IoPPN), King’s College London, London, UK; 2 Centre for Brain and Cognitive Development, Birkbeck, University of London, London, UK; 3 MRC Social, Genetic and Developmental Psychiatry Centre, IoPPN, King’s College London, London, UK; 4 NIHR Biomedical Research Centre at South London and Maudsley NHS Trust, London, UK; 5 Department of Psychology, IoPPN, King’s College London, London, UK; 6 Department of Biostatistics, IoPPN, King’s College London, London, UK; 7 Department of Psychological Sciences, Birkbeck, University of London, London, UK; 8 Department of Psychology, University of Southampton, Southampton, UK; 9 Department of Experimental Clinical and Health Psychology, Ghent University, Ghent, Belgium; 10 School of Psychology, University of East London, London, UK; 11 Department of Psychology, University of Cambridge, Cambridge, UK

## Erratum

The original publication [1] misses one author. The author details can be found below.

Dr. Luke Mason, Centre for Brain and Cognitive Development, Birkbeck, University of London, London, UK.
