# Supplementary material for: Attention training for infants at familial risk of ADHD (INTERSTAARS): study protocol for a randomised controlled trial
Source: Trials. 2016 Dec 28;17:608. doi: 10.1186/s13063-016-1727-0 (PMC5192597; doi:10.1186/s13063-016-1727-0)
Supplement: Additional file 1: Table S1. — Summary of measures. (PDF 99 kb) [file 13063_2016_1727_MOESM1_ESM.pdf]

Table 1. ***Summary of measures***

	**Measure**	**10-14 months Baseline lab visit**	**10-14 months Baseline home visit**	**12-16 months Inter - mediate home visit**	**10-18 months Intervention period**	**14- 18 months Outcome home visit**	**14-18 months Outcome lab visit**	**24 month follow up**	**36 month follow up**
**Parent questionnaires/ interviews**	Demographic Interview	X							
	Demographic questionnaire	X							
	Medical and psychiatric history interview	X							
	Medical and psychiatric history interview	X							
	Sleep and Settle Questionnaire	X					X		
	Visual Media Exposure History		X						
	Sleep Diary				X				
	Visual Media Diary				X				
	Infant Behaviour Questionnaire Revised	X		X			X		
	Vineland Adaptive Behaviour Scales	X					X	X	X

	Early Child Behavioural Questionnaire							X	X
	Child Behavioural Checklist Preschool								X
	Preschool Diagnostic Interview Schedule for Children								X
	Behavioural Rating Inventory of Executive Functions								X
**Observational assessments**	Toy Play	X	X	X		X	X	X	X
	Free Play	X					X	X	X
	Mullen Scales of Early Learning	X					X	X	X
	Early Social Communication Skills		X	X		X			
**Eye-tracking**	**Cognitive control task**	**X**	**X**	**X**		**X**	**X**		
	**Gap-overlap task**	**X**	**X**	**X**		**X**	**X**		
	Sequence learning task	X	X	X		X	X		

	**Sustained attention task**	**X**	**X**	**X**		**X**	**X**
	Visual paired comparison task	X	X	X		X	X
	Distractor task	X	X	X		X	X
**Neurophysiological**	EEG	X					X
	Autonomic control measures	X					X

***Measures that contribute to the primary outcome are shown in dark gray and bold font**; secondary outcomes shown in light gray.
